# An efficient single-arm Bayesian adaptive trial algorithm to evaluate de-intensified oncologic treatment

**DOI:** 10.1186/s13063-025-09315-6

**Published:** 2025-12-10

**Authors:** Yuan Zhong, Zeynep Baskurt, Mahmood Aminilari, Jennifer Seelisch, Lindsay A. Renfro, Sharon M. Castellino, Wei Xu, David Hodgson

**Affiliations:** 1 Biostatistics Department, University Health Network, Toronto, ON Canada; 2 Department of Radiation Oncology, Princess Margaret Cancer Centre, University Health Network, Toronto, ON Canada; 3 Children’s Hospital London Health Sciences Centre, Division of Hematology/Oncology, Western University, London, ON Canada; 4 Division of Biostatistics, University of Southern California, and Children’s Oncology Group, Los Angeles, CA USA; 5 Department of Pediatrics, Emory University School of Medicine, Atlanta, GA USA; 6 Aflac Cancer and Blood Disorders Center, Children’s Healthcare of Atlanta, Atlanta, GA USA; 7 Department of Biostatistics, Dalla Lana School of Public Health, University of Toronto, Toronto, ON Canada

**Keywords:** Bayesian adaptive trial, Single-arm trial, Multi-stage interim analysis, Bayesian statistics

## Abstract

**Background:**

In clinical trials, evaluating de-intensified oncologic treatment strategies can help reduce treatment-related toxicities while preserving patients’ quality of life. However, de-intensification is typically evaluated in cancers with a low relapse rate, and if the cancer type is uncommon, a randomized trial may require an impractically extended period to accumulate sufficient events for reliable inferential conclusions.

**Method:**

This paper introduces a Bayesian adaptive method for the single-arm trial design that provides efficient analysis of survival data under these constraints. By incorporating data from previous studies to establish prior knowledge and a historical control arm, this approach enables robust and accurate estimations and predictions for trial design, sample size determination, and inferential decision-making. To support the implementation of this method, we developed an R package called “BayesAT,” which offers significant flexibility in modelling and supports multi-stage interim analyses, particularly for evaluating de-intensified oncologic treatments.

**Result:**

Our approach is validated through comprehensive simulation studies and sensitivity analyses. Additionally, this algorithm has been applied to a pediatric Hodgkin lymphoma trial, showcasing its capability to effectively leverage information from previous studies and conduct interim analyses that expedite conclusions regarding treatment efficacy.

## Introduction

Oncology research frequently evaluates approaches that compare de-escalation treatment to the standard therapy, attempting to reduce treatment-related toxicities and enhance patient quality of life. For example, in the Children’s Oncology Group AHOD 0031 trial, there was no significant reduction in the event-free survival (EFS) or overall survival (OS) with the omission of radiation therapy for children with Hodgkin lymphoma who achieved a rapid and complete response to dose-dense chemotherapy [1]. Similarly, a non-inferiority trial involving 6711 breast cancer patients compared adjuvant chemoendocrine therapy with endocrine therapy alone and found that the omission of chemotherapy did not significantly compromise outcomes [2]. Furthermore, a randomized phase II trial in oropharyngeal cancer assessed reduced- or standard-dose postoperative radiation therapy in intermediate-risk patients [3], demonstrating that a de-intensification strategy resulted in equally favourable outcomes. These examples underscore the potential benefits of reducing treatment intensity in specific clinical settings.

While randomized control trials (RCTs) are the gold standard for assessing the safety and effectiveness of medical interventions, they have certain limitations. Reduction of treatment intensity is typically of clinical relevance when relapse risk (and hence event rate) is low, and only a small subset of patients is eligible for de-intensified treatment each year. As a result, the duration required to enroll sufficient patients and the subsequent follow-up can be prolonged to monitor study outcomes. Consequently, RCTs usually require extensive resources and can be infeasible to execute due to the complexity of design and rigorous regulatory requirements.

Recently, there has been increased interest in utilizing non-randomized trial designs in the context of conditional drug approvals [4, 5]. Compared to traditional designs, various data-driven approaches like Bayesian statistical methods, single-arm studies, and adaptive designs are increasingly used in clinical trials [6]. These alternatives follow specific guidelines and regulations, offering faster patient access to innovative treatments than traditional RCTs. Bayesian methods integrate prior research and expert opinion to enhance accuracy and efficiency in trial design and analysis [7, 8]. Single-arm designs are preferable for conditions with limited patient populations and well-documented histories, although they require meticulous historical control comparison [9–13]. Adaptive trials allow for pre-planned modifications based on interim data, enhancing flexibility, and efficiency while maintaining scientific validity [14–16].

In practical applications, utilizing the Bayesian method for adaptive trials can significantly boost decision-making efficiency compared to traditional frequentist methods [17, 18]. Several studies indicate that this design needs smaller sample sizes and fewer events, leading to quicker conclusions in trials [19, 20]. For example, Bayesian adaptive trials were extensively used during the COVID-19 pandemic to evaluate effective treatments, leveraging the ongoing accumulation of data to improve treatment strategies [21]. In addition, the Bayesian method allows for the analysis of historical controls through extensive simulation studies and the formulation of prior knowledge via sensitivity analysis [22–24]. The Bayesian method provides great flexibility as an alternative approach for clinical trial design, which can be applied to both adaptive trials and single-arm designs. Despite its advantages, the effectiveness of this approach depends heavily on the selection of priors and likelihood models. In addition, the Bayesian algorithm can be computationally intensive, and existing algorithms may not cover the full spectrum of clinical research needs [25]. Therefore, there is a significant need to develop Bayesian adaptive trial algorithms tailored to specific research requirements.

In this paper, we propose to use a Bayesian survival model (see Chapter 2 of [26]) in a single-arm adaptive trial. This method is particularly well-suited for the de-intensified designs in the context of infrequent outcome events, such as small patient populations with high cure rates and low event rates. Based on prior knowledge, the Bayesian survival model can effectively estimate hazard rates and predict survival probability for studies with low event rates. Moreover, this algorithm can be applied to generate pilot data and evaluate the potential efficacy or futility of a de-intensified treatment strategy before launching a randomized trial. The probabilistic results derived from Bayesian analysis provide robust, evidence-based scientific insights. To execute timely clinical trials, we have developed a Bayesian adaptive trial algorithm based on this survival model, which is capable of performing multiple-stage interim analyses. Unlike the two- or three-stage approach, we can implement Bayesian inference at each stage with updated interim data during both patient enrollment and early follow-up phases. Interim Bayesian inference can effectively utilize the posterior estimation to provide more flexible and prompt decisions for the sample size determination. With evidence of high efficacy during the interim analysis, we can conclude the effectiveness of the new treatment in a shorter time frame, allowing patients to reduce intensive treatment safely. In addition, we develop a computationally efficient algorithm for the Bayesian adaptive trial design, which is available as an R package called “BayesAT.” It offers various functions for users to conduct simulation studies, generate pilot data, and analyze real clinical data.

The outline of the paper is as follows. The “Methodology” section introduces the Bayesian survival model and sample size determination methodologies, including the Bayesian interim analysis and the proposed algorithm. In the “Simulation” section, we implement the proposed algorithm across various simulation studies. The “Data applications” section discusses the application of the Bayesian adaptive trial to a Hodgkin Lymphoma trial, demonstrating how this approach can potentially lead to early conclusions about treatment efficacy.

## Methodology

Suppose a clinical trial collects data $D_n$ from *n* patients. The data $D_n$ includes the follow-up time denoted by $T_n = (t_1, \cdots , t_i, \cdots , t_{n})$ and the censoring times $C_n = (c_1, \cdots , c_i, \cdots , c_{n})$ that is independent of $T_n$. In addition, the event of interest can be defined as the occurrence of death, disease progression, and relapse, which are binary outcomes $\Delta _n = (\delta _1, \cdots , \delta _i, \cdots , \delta _n)$. If the event was observed before the trial concluded $(t_i < c_i)$, $\delta _i = 1$, and $\delta _i = 0$ otherwise. The clinical data $D_n$ may include the enrollment time and other patients’ information, denoted by $X_n = (x_1, \cdots , x_i, \cdots , x_n)$.

We apply the exponential distribution exp$(\lambda )$ to model the likelihood of time-to-event outcomes,

1 $$\begin{aligned} \mathcal {L}(\lambda \vert T_n, \Delta _n) = \prod _{i=1}^n f(t_i \vert \lambda )^{\delta _i} S(t_i \vert \lambda )^{1-\delta _i}, \end{aligned}$$

where the density functions $f(t_i \vert \lambda )$ and survival functions $S(t_i \vert \lambda )$ are formulated as follows,

$$\begin{aligned} f(t_i \vert \lambda ) = \lambda \exp \{-\lambda t_i\}\ \text {and}\ S(t_i \vert \lambda ) = \exp \{-\lambda t_i\}. \end{aligned}$$

We employ a Gamma prior G$(\alpha , \beta )$ for parameter $\lambda \in \Lambda$, with the probability density

2 $$\begin{aligned} \pi (\lambda ) = \frac{\beta ^{\alpha - 1}}{\Gamma (\alpha )} \lambda ^{\alpha -1} \exp \{-\beta \lambda \}. \end{aligned}$$

Integrating the likelihood (1) and the prior (2), the joint model is given by,

$$\begin{aligned} Q(\lambda , T_n, \Delta _n) = \frac{\beta ^{\alpha - 1}}{\Gamma (\alpha )} \lambda ^{\alpha + \sum \nolimits _{i=1}^n \delta _i - 1} \exp \{ - (\beta + \sum \limits _{i=1}^n t_i) \lambda \}, \end{aligned}$$

and the corresponding posterior density $f_\pi (\lambda \vert T_n, \Delta _n)$ is derived as,

$$\begin{aligned} f_\pi (\lambda \vert T_n, \Delta _n) \propto \lambda ^{\alpha + \sum \nolimits _{i=1}^n \delta _i - 1} \exp \{ - (\beta + \sum \limits _{i=1}^n t_i) \lambda \}. \end{aligned}$$

By factorizing the normalized constant, we can show that

$$\begin{aligned} \lambda \vert T_n, \Delta _n \sim \ \text {Gamma}\ (\alpha + \sum \limits _{i=1}^n \delta _i , \beta + \sum \limits _{i=1}^n t_i). \end{aligned}$$

The posterior mean and variance can be given by,

$$\begin{aligned} \text {E}_{\Lambda }( \lambda \vert T_n, \Delta _n) & = \frac{\alpha + \sum \nolimits _{i=1}^n \delta _i}{\beta + \sum \nolimits _{i=1}^n t_i},\\ \text {Var}_{\Lambda }( \lambda \vert T_n, \Delta _n) & = \frac{\alpha + \sum \nolimits _{i=1}^n \delta _i}{(\beta + \sum \nolimits _{i=1}^n t_i)^2}. \end{aligned}$$

The hyperparameters $\alpha$ and $\beta$ are set based on extensive simulation studies and sensitivity analyses. In contrast to using a Gamma prior, we employ the Jeffreys prior [27], which is a non-informative prior as $\pi (\lambda ) \propto 1/\lambda$. The detailed prior elicitation is provided in the Appendix 1.

The analysis of the trial using Bayesian inference can estimate the posterior probability measure of $\lambda \ge r$, where *r* is a threshold:

$$\begin{aligned} \text {P}_{\Lambda }(\lambda \ge r \vert T_n, \Delta _n) = \int _{r}^{\infty } f_\pi (\lambda \vert T_n, \Delta _n) d\lambda . \end{aligned}$$

With a significant value $\alpha \in (0,1)$, we can conclude that the trial is successful if P$_{\Lambda }(\lambda \ge r \vert T_n, \Delta _n) \ge \alpha$. It is also known as the $(1-\alpha )100\%$ credible interval denoted by $(r, +\infty )$.

In addition, we can estimate the predictive measure for any new observations $D_{\text {new}} = ({T}_{\text {new}}, {C}_{\text {new}}, {\Delta }_{\text {new}})$ [28],

3 $$\begin{aligned} p_n = \int _{D_{\text {new}} } f_n(t) d t, \end{aligned}$$

where the density function of $f_n(t)$ is derived based on the posterior

$$\begin{aligned} f_n(t) = \int _{\lambda \in \Lambda } f(t\vert \lambda ) f_\pi (\lambda \vert T_n, \Delta _n) d \lambda . \end{aligned}$$

In clinical research, this predictive measure $p_n$ could represent metrics such as 2-year event-free survival (2-Y EFS) or 5-year event-free survival (5-Y EFS). Using the Bayesian predictive measure, the statistical hypothesis can be established as

4 $$\begin{aligned} \text {H}_0: p_n \le \eta \ \text {vs.}\ \text {H}_a: p_n> \eta . \end{aligned}$$

The threshold value $\eta$ can be derived from summary statistics of previous studies that applied the same inclusion and exclusion criteria. The historical control $\eta = p_0$ can be used to assess whether the treatment demonstrates superiority or non-superiority. This Bayesian predictive measure can also be flexibly expressed as an estimate of the hazard rate, and the formulation of the hypothesis 4 can be established under the proportional hazards framework [29, 30].

In addition, the test of statistics of this predictive measure can be used to determine the required sample size for clinical trials. The sample size can be the smallest number of observations based on probability criteria $\beta$ as follows,

5 $$\begin{aligned} {n}^* = \min \{ n \in \mathbb {N}:P(p_n \ge \eta ) \ge \beta \}, \end{aligned}$$

Unlike the frequentist approach, which usually relies on fixed sample sizes, the Bayesian method provides greater flexibility in determining sample size by incorporating historical data and prior beliefs. Especially, it allows the effect size in power calculations to be non-deterministic and enables dynamic monitoring and adjustments during the trial design process.

### Bayesian adaptive trials

In a Bayesian adaptive trial, the sample size can be determined based on the survival outcomes assessed through multiple-stage interim analysis. We conduct a *K*-stage interim analysis corresponding to *K* groups of patients and apply the Bayesian method throughout the trial.

In any *k*th stage data $D_n^{(k)}$, the follow-up times of patients in the group are denoted as $T^{(k)}_{n} = ( t^{(k)}_1, \cdots , t^{(k)}_{n_k})$, with the corresponding censoring times $C^{(k)}_{n} = ( c^{(1)}_1, \cdots , c^{(k)}_{n_k})$ and the event status $\Delta ^{(k)}_{n} = ( \delta ^{(1)}_1, \cdots , \delta ^{(k)}_{n_k})$. Information from earlier groups up to the $(k-1)$th stage can be consolidated as $T^{(1:k-1)}_n$, $C^{(1:k-1)}_n$, $\Delta ^{(1:k-1)}_n$. In the Bayesian adaptive framework, the posterior from the previous $(k-1)$th stage is used as the prior for the current *k*th stage,

$$\begin{aligned} \pi ^{(k)} (\lambda ) = f_{\pi }^{(k-1)} (\lambda \vert T^{(1:k-1)}_n,\Delta ^{(1:k-1)}_n). \end{aligned}$$

Each stage is analyzed with re-estimated prior, permitting ongoing adjustments to the parameter posterior $f_{\pi }^{(k)}(\lambda \vert T^{(1:k)}_n,\Delta ^{(1:k)}_n)$ based on the cumulative evidence. The predictive probability measure at each stage is given by

6 $$\begin{aligned} p_n^{(k)} = \int _{D_n^{(k)}}\int _{\lambda \in \Lambda } f(t \vert \lambda ) f_{\pi }^{(k)}(\lambda \vert T^{(1:k)}_n,\Delta ^{(1:k)}_n) d \lambda dt \end{aligned}$$

Through the interim analysis, the statistical hypothesis can be given by

$$\begin{aligned} \text {H}_0: p_n^{(k)} \le \eta \ \text {vs.}\ \text {H}_a: p_n^{(k)}> \eta . \end{aligned}$$

With a predetermined threshold $\eta$, clinicians can make decisions based on the results of the hypothesis test at each stage.

In addition, the required sample size can be adjusted based on whether the predictive probability reaches the threshold with stage-specific probability criteria $\beta _k$:

$$\begin{aligned} n^* = \min \left\{ n = \sum \limits _{k=1}^\kappa n_{k}: P(p_n^{(\kappa )}> \eta ) \ge \beta _k , \kappa = 1, 2, \cdots , K \right\} . \end{aligned}$$

Stage-specific probability criteria $\beta _k$ can incorporate an error-rate spending function to control false positive error [31, 32]. Various Bayesian sequential designs implement different types of decision rules to achieve this goal [17, 33, 34]. In the paper, each interim analysis set $\beta _k = 1 - \alpha *k/K$ for any $k = 1, \cdots , k$ and a predetermined significance level $\alpha$. Compared with the frequentist approach, which requires the sample size to be fixed before the trial design, the minimum required sample size $n^*$ can be adjusted based on the clinical performance.

### Algorithm

**Fig. 1:**
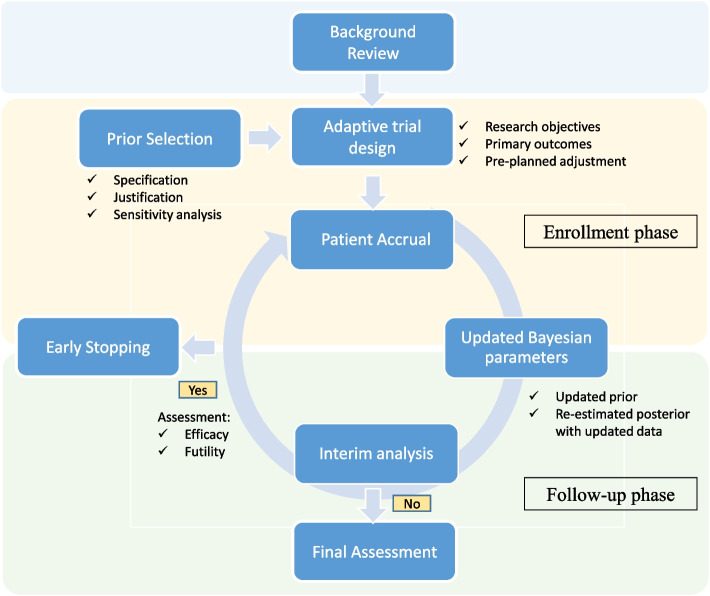
The single-arm Bayesian adaptive trial algorithm

We can develop a Bayesian adaptive trial algorithm to implement multiple-stage interim analysis shown in Fig. 1. Before implementing the algorithm, a background review and survey are necessary to design the clinical trial. For a single-arm trial, historical data surveys are essential to establish a reliable benchmark for analysis. To minimize potential sampling bias from historical studies, the study’s inclusion and exclusion criteria must be carefully evaluated. For example, propensity score matching on baseline characteristics can assess comparability and confirm that historical cohorts are sufficiently relevant to the current analysis [35, 36]. In addition, we utilize a comprehensive approach to prior elicitation through careful specification, justification and sensitivity simulations. The prior knowledge can be obtained from a diverse range of sources to enhance the accuracy and robustness of the Bayesian model.

Interim analyses are performed during both the enrollment and follow-up phases to adapt the trial based on real-time data. Unlike the fixed sample-size frequentist approach, we can implement multiple-stage interim analysis with updated data. Each stage of Bayesian inference is continually adjusted with the new information. Thus, the trial can be dynamically monitored against predetermined criteria for efficacy or futility and reach early termination if the thresholds are met.

Following the standards of Bayesian analysis [37], the methodology includes a thorough description of prior selection, the specific Bayesian inference method, and the interim analysis results. This comprehensive approach ensures that the trial design is both scientifically rigorous and highly adaptive to emerging data, ultimately aiming to enhance the efficiency and ethical conduct of clinical research.

## Simulation

In this section, we conduct different simulation studies to examine the Bayesian survival model performance and implement the proposed algorithm for the simulated clinical trial.

### Data generating process

**Fig. 2:**
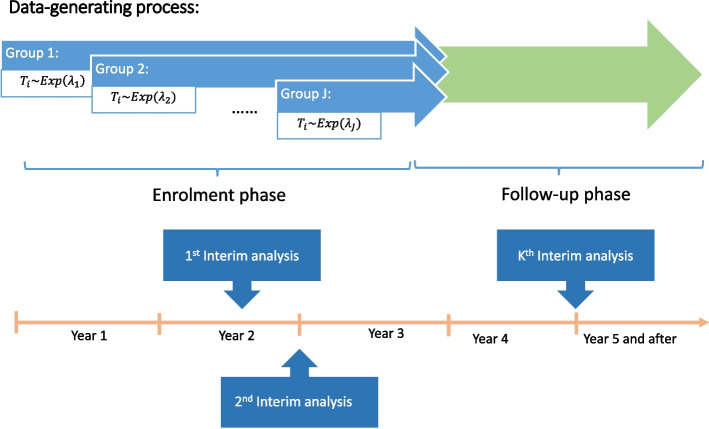
Diagram of data generating process and design of *K*-stage interim analysis

The generating data shown in Fig. 2 is designed as a clinical trial lasting up to 6 years with overall high survival rates and fewer events. Patient enrolment is scheduled every 4 months in the initial 3 years, resulting in $n_j$ observations for each enrollment group with $j = 1, 2, \cdots , 9$. The number of patients $n_k$ can be the same in each group or randomly simulated from a Poisson distribution with a specified mean $\mu _j$. For each group, the survival times of patients denoted by $T_j = (t_1, \cdots , t_{n_j})$ are independently and identically distributed samples drawn from an exponential distribution Exp$(\lambda _j)$, and the values of hazard rate $\lambda _j$ are sampled from the uniform distribution based on specific hypothesis. The occurrence of events that happened to patients with survival time less than the maximum duration within each group is simulated using a binomial distribution Binom$(n_j, p_j)$. The simulation studies set the event rate $p_j$ drawn from the uniform distribution. Given that the trial extends over 6 years, the survival times are truncated after the sixth year. With a 3-year enrollment period, a minimum 3-year follow-up time is designed in this simulation. Within the dataset of observations where no events have occurred, a $10\%$ censoring rate is applied to the follow-up time.

### Bayesian hypothesis tests

In this simulation, we perform Bayesian hypothesis testing and evaluate the Bayesian survival model performance based on a frequentist perspective. The simulation studies are conducted to assess type I error control and to perform power analyses for sample size determination. The simulation studies consider enrollment with an equal number of patients for each group. The simulation setups are shown in Table 1. For type I error control simulation, the data are generated under the null hypothesis H$_0$. The power analysis simulates survival data with small hazard rates and event rates to represent the alternative hypothesis H$_1$. The analysis focuses on a 2-year event-free survival (2-Y EFS) rate as the primary outcome. We set the historical control with 2-year EFS (EFS$_0$) as $92\%$ and $90\%$ under the null hypothesis in each scenario.

**Table 1:** Two simulation setups with different hazard rates,event rates, prior and null hypotheses

	Data generating		Prior setup	Historical control
	Hazard rate $\varvec{\lambda }_{\varvec{j}}$	Event rate $\varvec{p}_{\varvec{j}}$	Gamma mean	H_0_: 2-year EFS
Simulation 1: Type I error control				
Case I	Unif(0.041, 0.042)	Unif(0.22, 0.23)	Mean = 0.038	EFS$_0 =$ $92\%$
Case II	Unif(0.053, 0.054)	Unif(0.27, 0.28)	Mean = 0.048	EFS$_0 =$ $90\%$
Simulation 2: Power analysis				
Case I	Unif(0.02, 0.03)	Unif(0.07, 0.12)	Mean = 0.025	EFS$_0 =$ $92\%$
Case II	Unif(0.03, 0.04)	Unif(0.10, 0.15)	Mean = 0.033	EFS$_0 =$ $90\%$

The estimated $\hat{\text {EFS}}$ is compared EFS$_0$, and the hypotheses are formulated as follows,

$$\begin{aligned} \text {H}_0: \hat{\text {EFS}} < \text {EFS}_0\ \text {vs.}\ \text {H}_1: \hat{\text {EFS}} \ge \text {EFS}_0. \end{aligned}$$

Under null hypothesis, survival outcomes fall below the historical control EFS$_0$, whereas the alternative hypothesis suggests that the EFS meets or exceeds the target EFS.

**Fig. 3:**
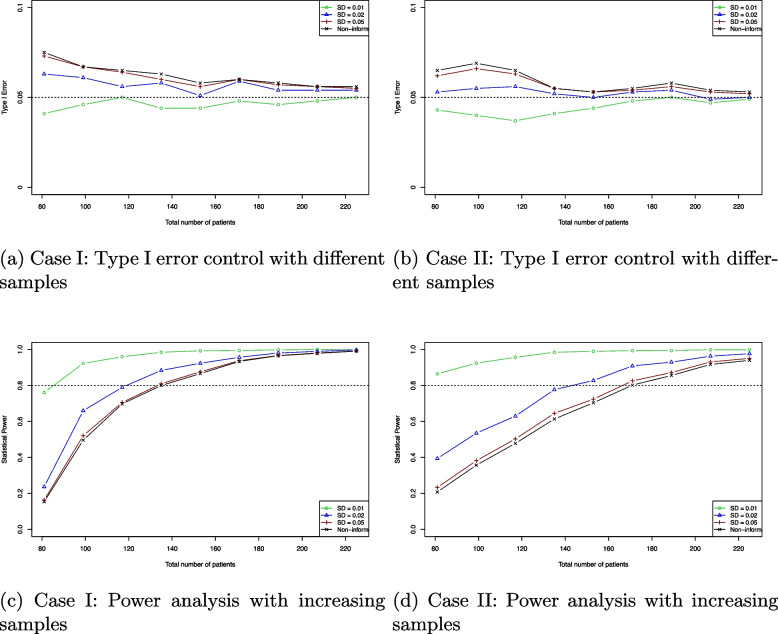
Type I error control and power analysis results for patient sample sizes ranging from 80 to 225 with different prior functions. As sample sizes increase, the empirical type I error converges, and the estimated statistical power gets larger. **a** and **b** represent Case I and Case II for type I error control, and **c** and **d** represent Case I and Case II for power analysis in Table 1, respectively. The standard deviations (SD) of the Gamma priors represent varying levels of precision: strongly informative (SD = 0.01), moderately informative (SD = 0.02), and weakly informative (SD = 0.05). The non-informative prior (Non-inform) is derived from Jeffreys’ prior

This study conducts $M=1000$ independent simulations using the proposed Bayesian survival model. Gamma priors are used for the hazard rate $\lambda _k$, with informative priors based on different precision levels of the Gamma distribution and a non-informative prior derived from Jeffreys prior [27]. The test of statistics for type I error is defined as:

$$\begin{aligned} T_i = \left\{ \begin{array}{ll} 1,& \text {if}\ \text {P}(\hat{\text {EFS}} \ge \text {EFS}_0\vert \text {H}_0) \ge 95\% \\ 0,& \text {if}\ \text {P}(\hat{\text {EFS}} \ge \text {EFS}_0\vert \text {H}_0) < 95\%, \end{array}\right. \end{aligned}$$

and the empirical type I error rates are calculated as $T = (1/M)\sum \nolimits _{i=1}^M T_i$. Figure 3 shows that the Bayesian survival model can approximately achieve the nominal $5\%$ type I error. When using a strongly informative prior, the empirical type I error consistently falls below $5\%$. As the sample size increases, the estimates across different priors converge to the nominal level. It indicates that the selection of the prior function is critical for the Bayesian survival model.

In power analysis, the test of statistics is given by,

$$\begin{aligned} U_i = \left\{ \begin{array}{ll} 1, & \text {if}\ \text {P}(\hat{\text {EFS}} \ge \text {EFS}_0\vert \text {H}_1) \ge 95\% \\ 0, & \text {if}\ \text {P}(\hat{\text {EFS}} \ge \text {EFS}_0\vert \text {H}_1) < 95\%. \end{array}\right. \end{aligned}$$

**Fig. 4:**
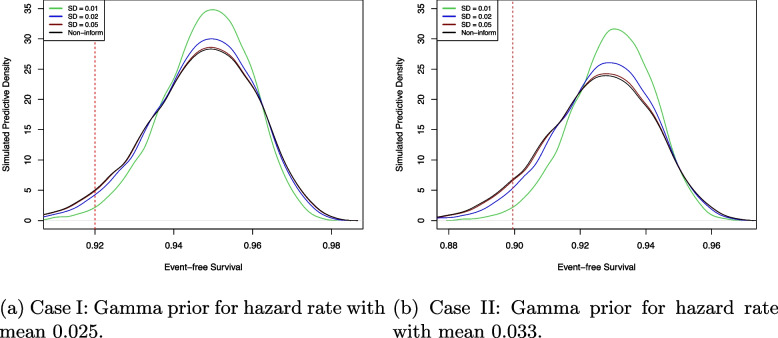
Posterior density plots based on 1000 replication simulations. **a** and **b** represent Case I and Case II in Table 1, respectively. The standard deviations (SD) of the Gamma prior reflect different levels of precision: strongly informative (SD = 0.01), moderately informative (SD = 0.02), and weakly informative (SD = 0.05). The non-informative prior (Non-inform) is based on Jeffreys’ prior

The empirical power is calculated by $U = (1/M)\sum \nolimits _{i=1}^M U_i$. In Fig. 3, the proposed model achieves over $80\%$ power with a smaller sample size compared to the non-informative prior. The strongly informative prior (SD = 0.01) requires fewer than 100 subjects, while moderately informative priors (SD = 0.02) need 10 to 30 fewer subjects to reach $80\%$ power. No significant difference is observed between weakly informative and non-informative priors. Appendix 2 presents additional simulation results. Table 3 in Appendix 2 provides reference sample size estimates obtained using the BayesDesign R package and PASS software. The average number of events across different samples are illustrated in Table 4 in Appendix 2. For the power analysis simulations, Fig. 4 illustrates the posterior density plots for 2-year EFS based on the standard deviations of the priors. The posterior density associated with the strongly informative prior (green) exhibits a more pronounced central tendency and light tails compared to the others.

### Interim analysis

This section demonstrates the proposed Bayesian adaptive trial algorithm through the six-stage interim analysis. The simulation studies design four scenarios with different parameters for data-generating, prior function setups, and historical control rates shown in Table 2. We simulate trial data sets with each case’s specific hazard rate $\lambda _j$, which can provide high survival probabilities. The low event rates are set through two scenarios, where $p_j$ is simulated from a Unif(0.05, 0.10) or Unif(0.10, 0.15). Based on the data generating process in the “Data generating process” section, the number of patient enrolments $n_j$ is first simulated from a Poisson distribution Pois$(\mu _j)$ with the mean rate $\mu _j = 10$. To ensure each stage with sufficient samples, a minimum of 6 patients per enrollment period is maintained. Over a 3-year design, this simulation yields an average total enrollment of approximately 100 patients.

**Table 2:** Four simulation setups for interim analysis with different hazard rates, event rates, prior and null hypotheses

Simulation	Data generating		Prior setup	Historical control
	Hazard rate $\varvec{\lambda }_{\varvec{j}}$	Event rate $\varvec{p}_{\varvec{j}}$	Gamma mean	H_0_: 2-year EFS
Case I	Unif(0.02, 0.03)	Unif(0.05, 0.10)	Mean = 0.025	EFS$_0 = 92\%$
Case II	Unif(0.03, 0.04)	Unif(0.10, 0.15)	Mean = 0.035	EFS$_0 = 90\%$
Case III	Unif(0.04, 0.05)	Unif(0.05, 0.10)	Mean = 0.045	EFS$_0 = 94\%$
Case IV	Unif(0.06, 0.07)	Unif(0.10, 0.15)	Mean = 0.065	EFS$_0 = 92\%$

Interim analyses begin at the 18th month and continue every 6 months until the 48th month. The first analysis evaluates data with sufficient follow-up time, while subsequent analyses update survival data and apply Bayesian inference to new data. The hypothesis testing is based on a single-arm trial design. For $k = 1, 2, \cdots , 6$, the algorithm estimates $\hat{\text {EFS}}_k$ using updated priors from previous stages and historical data. The *z*-score boundaries for efficacy and futility are determined using discrete sequential boundaries with significance level $5\%$. The test statistics are calculated using the standardized *z* score.

$$\begin{aligned} \hat{z}_k = \frac{ \hat{\text {EFS}}_k - \text {EFS}_0}{\hat{\sigma }({\text {EFS}}_k)}, \end{aligned}$$

where $\hat{\sigma }({\text {EFS}}_k)$ is the posterior standard deviation. At each stage, the estimated *z*-score $\hat{z}_k$ is compared with the nominal significance levels $\alpha _k$, which is the predetermined criterion for each stage of the interim analysis. The efficacy hypothesis can be set as

$$\begin{aligned} \text {H}_0: \hat{z}_k < \alpha _k\ \text {vs.}\ \text {H}_1: \hat{z}_k \ge \alpha _k. \end{aligned}$$

**Fig. 5:**
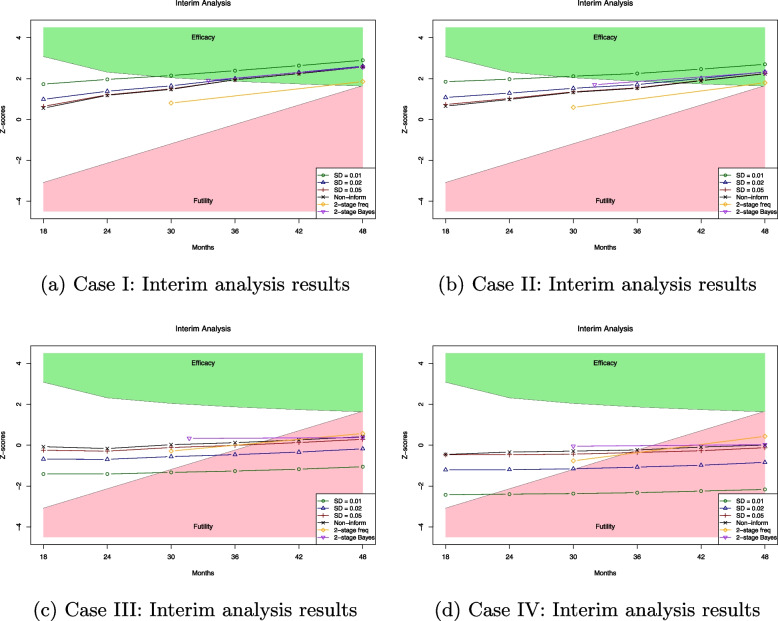
Interim analysis plot. The *z*-scores represent the median values at each stage over 1000 independent simulation results. Four scenarios are outlined in Table 2. The standard deviations (SD) of the Gamma prior reflect different levels of precision: strongly informative (SD = 0.01), moderately informative (SD = 0.02), and weakly informative (SD = 0.05). The non-informative prior (Non-inform) is based on Jeffreys’ prior [27]. A two-stage frequentist approach and two-stage Bayesian approach are used for comparison

In the simulation studies, the proposed Bayesian methods are compared with two-stage frequentist and Bayesian approaches:

- Two-stage frequentist design: The OneArm2stage R package [29] was used to implement the one-sample log-rank (OSLR) test. An interim analysis was scheduled at 30 months, followed by the final analysis at 48 months.
- Two-stage Bayesian design: The BayesDesign R package [30] was applied to conduct a two-stage Bayesian interim analysis. Unlike the frequentist approach, the timing of the interim analysis was not fixed but determined adaptively based on the transformed observation time [30]. The average time point for the first stage among 1000 simulation studies is presented in Fig. 5. The *z-*score is calculated based on the Bayesian survival model with a non-informative prior.

In Fig. 5, the Bayesian adaptive algorithm improves the efficiency of clinical trials by potentially reducing the trial duration. Models employing moderately and weakly informative priors demonstrate performance comparable to the non-informative approach. For instance, in Case I, an early stopping criterion based on efficacy could be met around the 30th month when using strongly informative priors, and around the 42nd month when using other priors. Detailed information regarding the month in which the stopping criterion is reached can be found in the Supplementary file Table 5 in Appendix 3. Compared with both the two-stage frequentist and Bayesian designs, the proposed adaptive approach offers a more efficient decision-making process and enables trials to reach conclusions within a shorter timeframe. The simulation studies conclude that the proposed Bayesian approach can be more robust based on careful prior elicitation and provide higher practicality by utilizing more flexible and timely analysis.

## Data applications

**Fig. 6:**
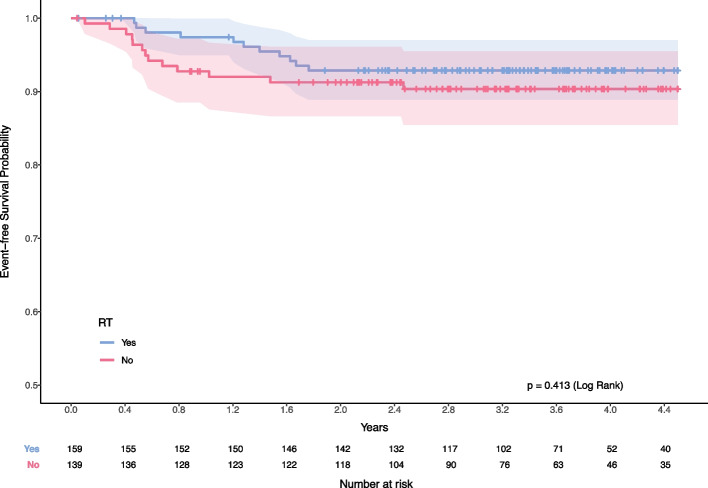
The Kaplan-Meier curve illustrates the event-free survival probabilities for pediatric Hodgkin lymphoma patients [38]

We examine the performance of the proposed Bayesian adaptive algorithm by implementing it in a previous randomized clinical trial on pediatric Hodgkin lymphoma [38] comparing two different chemotherapy regimens. There were 298 patients who received multi-agent chemotherapy in the superior arm of this trial, with radiation therapy given by non-random assignment to 159 of these patients, while 139 patients were treated with chemotherapy alone. As shown in Fig. 6, the 2-year event-free survival rate among all 298 patients in the superior treatment arm was $92.5\%$ ($95\%$ confidence interval (CI) 88.8 to 95.0, and was $91.2\%$ ($95\%$ confidence interval [CI], 84.9 to 94.9) among those receiving chemotherapy alone, and $93.6\%$ ($95\%$ confidence interval (CI), 88.5 to 96.5) among those receiving both chemotherapy and radiation. Given the recognized delayed toxicity of radiation therapy, one approach to improving long-term outcomes advocated by many clinical experts is the omission of radiation therapy, although due to the relative rarity of childhood HL and the low event rate, a second randomization to evaluate the impact of chemotherapy +/− radiation therapy was not practical. Thus, we can apply the proposed Bayesian adaptive algorithm to address this problem.

In this analysis, we apply the proposed Bayesian adaptive algorithm to estimate the 2-year event-free survival (EFS) rate for the de-intensified treatment group:

$$\begin{aligned} \text {H}_0: \hat{\text {EFS}}_k < \text {EFS}_0\ \text {vs.}\ \text {H}_1: \hat{\text {EFS}}_k \ge \text {EFS}_0. \end{aligned}$$

As an illustration of data application, the null hypothesis set EFS$_0 = 88.8\%$, which corresponds to the lower bound of the $95\%$ CI for the 2-year EFS among patients receiving multi-agent chemotherapy in the trial’s superior arm. Clinical experts considered this value the minimum acceptable 2-year EFS, reflecting the trade-off of omitting radiation therapy to potentially reduce late adverse effects. Thus, the standardized *z-*score can be given by,

7 $$\begin{aligned} \hat{z}_k = \frac{ \hat{\text {EFS}}_k - \text {EFS}_0}{\hat{\sigma }({\text {EFS}}_k)}, \end{aligned}$$

By applying multiple-stage interim tests, the inference can demonstrate whether the therapy without radiation can be considered acceptable compared to the combined chemotherapy and radiation.

**Fig. 7:**
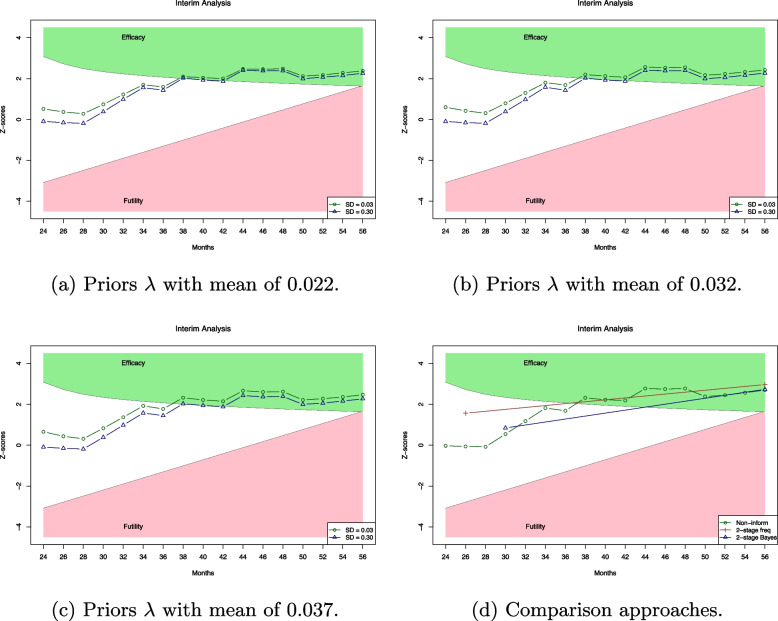
Interim analysis plot for the Hodgkin lymphoma trial [38]. The *z*-scores are calculated based on (7) over 17 stages. Plots **a**, **b**, and **c** illustrate the interim analysis using different Gamma prior functions, while plot **d** presents the results from the non-informative prior, two-stage frequentist approach, and two-stage Bayesian approach

From previous studies, the 2-year EFS difference between the RT and non-RT arm is approximately up to $4\%$, and the hazard ratios (HR) range between 0.7 and 1.5 [38–40]. Thus, we utilize the summary statistics to model different mean and standard deviation levels for the prior probabilities. Figure 7a–c show that the trial meets the efficacy criterion at the 38th month for all selected priors. This suggests that the proposed model is robust across different prior functions, allowing for the conclusion that the therapy’s effectiveness without radiation can be determined within a shorter patient enrollment and follow-up period. The proposed Bayesian algorithm allows the trial design to make timely and reliable conclusions about the efficacy of the treatment.

For comparison, we evaluate the Bayesian adaptive trial with a non-informative prior, a two-stage frequentist design, and a two-stage Bayesian design. As shown in Fig. 7d, the analysis based on the non-informative prior provides similar interim analysis conclusions as the proposed Bayesian approach with a weakly informative prior, which can stop the trial after the completion of patient accrual. Two-stage design is set with the first analysis before the 32nd month. Figure 7d indicates that the two-stage design, with its first analysis scheduled before month 32, shows that both the Bayesian and frequentist approaches fail to reach a conclusive interim decision.

## Discussion

This study proposed and validated a Bayesian adaptive trial design using a survival model, particularly in situations where traditional randomized control trials (RCTs) are not feasible. By leveraging a Bayesian framework, our study could address several limitations inherent to classical fixed-sample designs, providing a flexible and dynamic approach to single-arm clinical trial design and analysis. The methodology was validated through comprehensive simulation studies and applied to a pediatric Hodgkin lymphoma trial, demonstrating the algorithm’s capability to advance conclusions about treatment efficacy. The results highlight the robustness of this approach in inferring treatment efficacy, particularly with small sample sizes and available historical data. Applying this methodology to a pediatric Hodgkin lymphoma trial demonstrates its potential to expedite conclusions regarding treatment efficacy, especially in oncology, where prompt and precise assessment of new treatments is vital. This underscores the value of Bayesian methods in clinical research.

The integration of our findings with existing research highlights the broad applicability and effectiveness of Bayesian adaptive trial designs. The ability to conduct multistage interim analyses and update trial parameters dynamically ensures that the trial designs remain both efficient and valid. Our study contributes to the growing body of evidence that supports the use of Bayesian methods in clinical trials, particularly for conditions involving small populations or rare diseases. Furthermore, the use of historical controls and prior knowledge, as demonstrated in our methodology, addresses one of the significant challenges in clinical research: the recruitment of sufficient participants. By leveraging existing data, Bayesian adaptive designs can help overcome this hurdle, making it feasible to conduct robust trials even in challenging scenarios.

The advantages observed in this study are consistent with findings from similar research in the field. Several studies have highlighted the efficiency benefits of Bayesian adaptive designs [15, 41]. In the context of oncology, where our methodology was applied, the Bayesian adaptive designs have shown promising results. For instance, a study proposed an innovative Bayesian adaptive platform trial design for lymphoma, aiming to reduce the cost and ethical risks associated with traditional clinical trials [42]. By borrowing adaptively from historical control data, the design optimizes the randomization ratio for novel treatments when interim analysis indicates the comparability of control groups. This approach accelerates the introduction of new treatments while minimizing the number of patients needed for the current study, making it both ethical and economical [42].

Ryan et al. [19] revealed that the Bayesian sequential approach could reduce sample size and lead to earlier trial completion without compromising the study’s conclusions. They suggested that Bayesian adaptive designs have the potential to improve the efficiency and cost-effectiveness of phase III clinical trials in critical care. Berry et al. [17] discussed the use of Bayesian adaptive methods in the context of clinical trials, emphasizing their ability to incorporate prior information and adapt based on accumulating data, thus improving the decision-making process in trials. Similarly, in a three-arm exercise trial for breast cancer patients undergoing chemotherapy, a Bayesian-adaptive decision-theoretic approach substantially reduced the required sample size. This approach identified the most effective intervention after analyzing data from only 72 patients, compared to a frequentist approach that would have required data from 180 patients for a similar conclusion [43]. The success of the I-SPY 2 trial in identifying novel drugs that are biologically active against cancer also highlights the potential for adaptive platform trials to drive innovation in oncology research [44].

Our work extends the application of Bayesian methods by focusing on a specific survival model within the single-arm trial design. This approach is particularly relevant for diseases with de-intensified treatment strategies, such as childhood leukemia and lymphoma, where patient populations are relatively small, event rates are low, and historical data are available. In this study, we primarily evaluate treatment efficacy in trials with a time-to-event response as the primary outcome. To further investigate de-intensified treatment studies, the current Bayesian adaptive trial framework can incorporate composite clinical outcomes by combining time-to-event endpoints with patients’ quality of life and health utility scores based on [45, 46].

### Strengths and limitations of the study

One of the strengths of our study is the comprehensive validation of the proposed methodology through extensive simulation studies and its application to a real clinical trial. This dual approach ensures that the theoretical benefits of the Bayesian adaptive design are backed by empirical evidence, enhancing the credibility and applicability of our findings. Moreover, the use of a Bayesian framework allows for continuous learning and adaptation throughout the trial, which is a considerable advantage over the fixed-sample designs. This adaptability not only improves the efficiency of the trial but also ensures that the conclusions drawn are based on the most current and comprehensive data available.

Despite the promising results, our study is not without limitations. One of the primary challenges in the single-arm trial design is to balance the patients’ characteristics in the current trial and historical cohorts. The heterogeneity between two cohorts or biased sample selection can compromise the validity of the analysis, leading to biased estimation and incorrect clinical decisions. Thus, the inclusion and exclusion criteria should be carefully constructed to ensure comparability between patients across studies. Prior to applying the proposed method, an assessment of patients’ baseline characteristics using propensity scores should be conducted to evaluate the similarity between the trial and historical cohorts [35, 36]. The major challenge in Bayesian model is the selection of appropriate prior distributions. Inaccurate or biased priors can significantly impact the outcomes, potentially leading to erroneous conclusions. While our study employed robust methods for prior selection, such as the Bayes factor estimation [47], this remains a critical area for further research. Additionally, the computational complexity associated with Bayesian methods can hinder their widespread adoption. Implementing and running these trials require sophisticated statistical software, which may not be readily available in all research settings. Another limitation is the potential for variability in historical data, which can affect the reliability of the Bayesian model. Ensuring the quality and relevance of historical controls is essential for the success of the Bayesian approach. In practice, the interim-analysis design and its implementation should be developed in close collaboration with clinicians and other domain experts. Misspecified algorithm parameters, such as priors, analysis timelines, or error-spending rules, can affect operating characteristics, inflate the false-positive rate, and ultimately lead to erroneous decisions. Our proposed thresholding rule is intuitive and designed to gradually relax decision criteria across interim analyses. However, it hasn’t been formally calibrated or validated in the literature [17, 33, 34]. In future work, we plan to evaluate the operating characteristics of the full adaptive design, such as type I error and power, through simulations or analytical methods, and continue improving the R package accordingly.

## Conclusion

The study demonstrates the significant potential of Bayesian adaptive trials to enhance the efficiency and robustness of clinical research, particularly in scenarios where traditional RCTs are not feasible. By leveraging a Bayesian framework, the proposed methodology offers substantial flexibility in modelling and data-driven inference, facilitating robust sample size determination and enabling multi-stage interim analyses. While challenges remain, particularly in prior selection and computational demands, the advantages of Bayesian adaptive designs in advancing conclusions and integrating prior knowledge make them a promising alternative to conventional trial designs.

## Figures and Tables

**Fig. 8:**
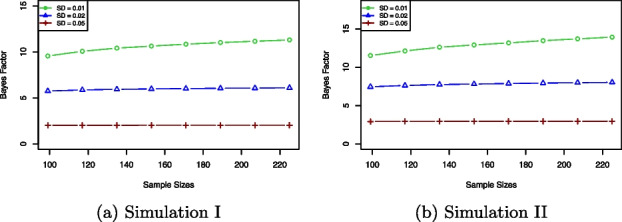
Estimated Bayes factor for the chosen priors over the non-informative prior in the power analysis

## Data Availability

N/a.

## Appendix 1: Prior elicitation

The prior selection is crucial for Bayesian inference. The proposed non-informative prior in this paper is established based on Jeffreys prior [27]. Through the Bayesian estimation method, we can conclude the posterior density is given by

$$\begin{aligned} f_{\pi _0}(\lambda \vert T_n, \Delta _n) \propto \lambda ^{\sum \nolimits _{i=1}^n \delta _i -1}\exp \{ -\lambda \sum \limits _{i=1}^n t_i\}. \end{aligned}$$

The posterior mean is $\sum \nolimits _{i=1}^n \delta _i/\sum \nolimits _{i=1}^n t_i$, which is identical to the maximum likelihood estimation (MLE) from the frequentist approach.

We can use Jeffreys prior as the reference level to choose the informative Gamma priors through sensitivity tests with different hyperparameters $\alpha$ and $\beta$. As illustrated in Fig. 8, the Bayes factor compares the priors used in our simulation with Jeffreys’ prior. According to the criterion, Bayes factor values less than 3.2 suggest that the data provides similar support for both the weakly informative prior with a standard deviation of 0.05 and Jeffreys’ prior. This observation is consistent with the posterior estimates shown in Fig. 4, where the weakly informative prior results in a density curve comparable to that from the non-informative prior. With a smaller standard deviation in Gamma prior, the difference between informative prior and Jeffreys prior becomes more significant. Thus, we categorize the priors used into three levels based on their standard deviations: strongly, moderately, and weakly informative for our analysis.

## Appendix 2: Power analysis results

In addition to Fig. 3, we present further details of the simulation results. Table 3 summarizes the sample size estimates obtained from alternative methods. The average 2-year EFS derived from the simulated data was used as the alternative hypothesis. Using the BayeDesign R package, the simulated sample sizes were consistent with those reported in Table 3. For comparison, we also calculated the sample size under the same settings using a frequentist approach implemented in PASS 2025. Table 4 illustrates the average number of events generated over 1000 independent simulations.

**Table 3:** The sample size determination based on BayesDesign R package (the prior with a location parameter a = 1) and frequentist approach using PASS 2025

Power analysis						
Power	80%	85%	90%	80%	85%	90%
	BayesDesign			Frequentist method		
Case I	144	166	210	179	208	248
Case II	130	160	191	161	187	223

**Table 4:** The average number of events generated over 1000 simulations for simulation studies in Table 1

Sample size	81	99	117	135	153	171	189	207	225	Avg rate
Simulation 1: Type I error control										
Case I	18	22	26	30	34	38	42	46	50	$22\%$
Case II	22	27	32	37	42	47	52	57	62	$27\%$
Simulation 2: Power analysis										
Case I	13	16	19	22	24	27	30	32	35	$16\%$
Case II	11	12	14	16	18	20	22	24	26	$12\%$

## Appendix 3: Interim analysis simulation results

In this section, Table 5 shows the average months of interim analysis reaching the efficacy or futility stopping criterion over 1000 independent simulations.

**Table 5:** The interim analysis has reached the stopping criterion for the months

The month of interim analysis stops					
Prior	SD	Case I	Case II	Case III	Case IV
Strong	0.01	30	30	30	24
Moderate	0.02	36	42	36	30
Weak	0.05	36	42	36	36
Non-informative		36	42	36	36

## Materials availability

N/a.

## Code availability

R package and code are available from BayesAT.
